# 2,2′-[4-Acetyl-1,3-phenyl­enebis(­oxy)]diacetic acid

**DOI:** 10.1107/S1600536812047897

**Published:** 2012-11-28

**Authors:** Jian-Guo Wang, Ping Yan, Chan-juan Zhong, Guo-zhen Yu

**Affiliations:** a School of Chemistry & Environmental Engineering, Jiujiang University, Jiujiang 332005, People’s Republic of China

## Abstract

In the title compound, C_12_H_12_O_7_, the dihedral angles between the benzene ring and the mean planes of the 3-carb­oxy­meth­oxy, 1-carb­oxy­meth­oxy and acetyl substituents are 8.67 (7), 7.81 (6) and 10.3 (18)°, respectively. In the crystal, mol­ecules are linked by typical carb­oxy­lic acid O—H⋯O hydrogen bonds, forming a zigzag chain. C—H⋯O inter­actions also occur.

## Related literature

For a related structure, see: Zhang *et al.* (2007 ▶).
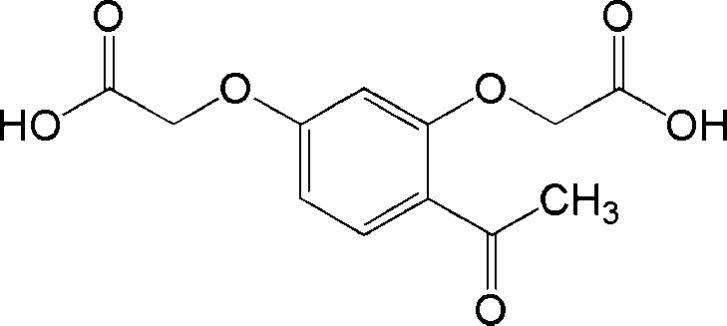


## Experimental

### 

#### Crystal data

C_12_H_12_O_7_
*M* *_r_* = 268.22
Triclinic, 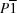

*a* = 5.1351 (6) Å
*b* = 7.8346 (9) Å
*c* = 15.6157 (18) Å
α = 86.217 (2)°
β = 81.321 (1)°
γ = 72.101 (2)°
*V* = 590.86 (12) Å^3^
*Z* = 2
Mo *K*α radiation
μ = 0.13 mm^−1^
*T* = 298 K
0.12 × 0.10 × 0.10 mm

#### Data collection

Bruker APEXII CCD diffractometer
Absorption correction: multi-scan (*SADABS*; Sheldrick, 2003 ▶) *T*_min_ = 0.985, *T*_max_ = 0.987
3902 measured reflections
2049 independent reflections
1860 reflections with *I* > 2σ(*I*)
*R*_int_ = 0.016

#### Refinement

*R*[*F* ^2^ > 2σ(*F* ^2^)] = 0.036
*wR*(*F* ^2^) = 0.120
*S* = 1.05
2049 reflections
176 parameters
H-atom parameters constrained
Δρ_max_ = 0.24 e Å^−3^
Δρ_min_ = −0.23 e Å^−3^

### 

Data collection: *APEX2* (Bruker, 2004 ▶); cell refinement: *SAINT* (Bruker, 2004 ▶; data reduction: *SAINT*; program(s) used to solve structure: *SHELXS97* (Sheldrick, 2008 ▶); program(s) used to refine structure: *SHELXL97* (Sheldrick, 2008 ▶); molecular graphics: *SHELXTL* (Sheldrick, 2008 ▶); software used to prepare material for publication: *SHELXTL* and *PLATON* (Spek, 2009 ▶).

## Supplementary Material

Crystal structure: contains datablock(s) I, global. DOI: 10.1107/S1600536812047897/go2075sup1.cif

Structure factors: contains datablock(s) I. DOI: 10.1107/S1600536812047897/go2075Isup2.hkl

Supplementary material file. DOI: 10.1107/S1600536812047897/go2075Isup3.cml

Additional supplementary materials:  crystallographic information; 3D view; checkCIF report

## Figures and Tables

**Table 1:** Hydrogen-bond geometry (Å, °)

*D*—H⋯*A*	*D*—H	H⋯*A*	*D*⋯*A*	*D*—H⋯*A*
O1—H1⋯O2^i^	0.82	1.83	2.6530 (14)	180
O6—H6⋯O5^ii^	0.82	1.83	2.6352 (15)	167
C2—H2⋯O7^iii^	0.93	2.44	3.3566 (17)	168
C4—H4⋯O6^iv^	0.93	2.52	3.4392 (19)	169

Symmetry codes: (i) 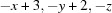; (ii) 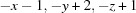; (iii) 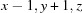; (iv) 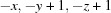.

**Fig. 1.**
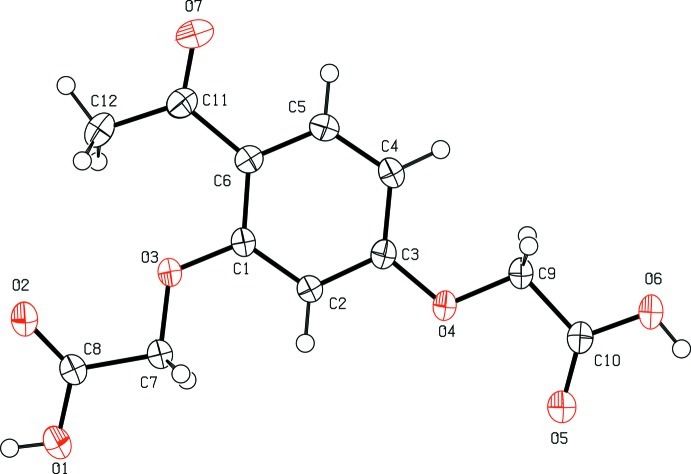
View of the molecule of (I) showing the atom-labelling scheme. Displacement ellipsoids are drawn at the 50% probability level.

**Fig. 2.**
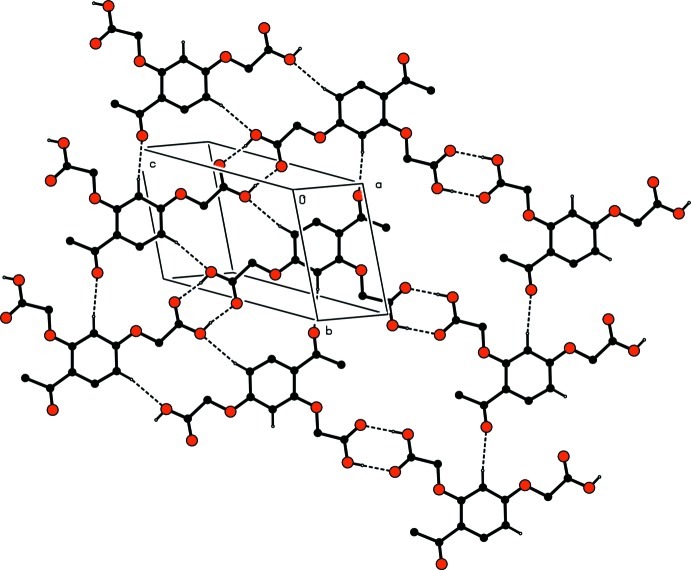
The crystal packing for (I), with hydrogen bonds shown as dashed lines. Hydrogen atoms not involved in the hydrogen bonding have been omitted.

## supplementary crystallographic information

### Comment

The title compound is a potential inhibitor of mushroom tyrosinase.

The dihedral angles between the mean planes of the benzene ring and those of the 2-carboxymethoxyl,(O1-C8-C7-O3-O2), 4-carboxymethoxyl (O6-C10-C9-O4-O5), and acetyl, (C11-C12-O7), substituents are 8.67 (7)°, 7.81 (6)°, and 10.3 (18)° respectively.

In the crystal, the molecules are linked by typical carboxylic acid O—H···O hydrogen bonding forming a one-dimensional zig-zag chain Table 1, Figure 2.

This chain is linked to anti-parallel chains by C—H···O weak hydrogen bonds to form a two dimensional sheet stabilizing the supramolecular structure, Table 1, Figure 2.

### Experimental

2,4-dihydroxyacetophenone (10.64 g, 0.07 mol) and potassium hydroxide (10.58 g, 0.189 mol) were dissolved in dry acetone (100 ml) in a three-neck flask. Then ethyl bromoacetate (28.06 g, 0.168 mol) was dropwise added at room temperature and vigorously stirred for 3 h. The progress of the reaction was monitored by TCL (Si gel, developing solvent *V* (acetone) /*V* (petroleum ether) = 1:2). After suction filtration and distillation to remove the solvent, a white solid was obtained, 19.76 g, yield 87.1%. This solid was dissolved in acetone (30 ml) and 20% aq. NaOH (50 ml) was added. The reaction mixture was stirred at 323 K for 1.5 h. After acidifying the misxture with dilute hydrochloric acid to pH=3, followed by suction filtrationand washing the residue with water, the target product was prepared. After recrystallization from ethanol, crystalline colorless needles were obtained.

### Refinement

All the carbon-bounded hydrogen atoms were located at their ideal positions with the C—H=0.93 Å, C—H=0.96 Å, C—H=0.97 Å and *U*_iso_(H)=1.2*U*_eq_(C). All the hydrogen atoms bonded to the oxygen atoms were located from the difference maps and refined with the restraints of O—H=0.82 (1) Å and *U*_iso_(H)=1.5*U*_eq_(O).

### Crystal data

**Table:**

C_12_H_12_O_7_	*Z* = 2
*M**_r_* = 268.22	*F*(000) = 280
Triclinic, *P*1	*D*_x_ = 1.508 Mg m^−^^3^
Hall symbol: -P 1	Mo *K*α radiation, λ = 0.71073 Å
*a* = 5.1351 (6) Å	Cell parameters from 3359 reflections
*b* = 7.8346 (9) Å	θ = 2.6–31.8°
*c* = 15.6157 (18) Å	µ = 0.13 mm^−^^1^
α = 86.217 (2)°	*T* = 298 K
β = 81.321 (1)°	Block, colourless
γ = 72.101 (2)°	0.12 × 0.10 × 0.10 mm
*V* = 590.86 (12) Å^3^	

### Data collection

**Table:**

Bruker APEXII CCD diffractometer	2049 independent reflections
Radiation source: fine-focus sealed tube	1860 reflections with *I* > 2σ(*I*)
Graphite monochromator	*R*_int_ = 0.016
φ and ω scans	θ_max_ = 25.0°, θ_min_ = 1.3°
Absorption correction: multi-scan (*SADABS*; Sheldrick, 2003)	*h* = −6→6
*T*_min_ = 0.985, *T*_max_ = 0.987	*k* = −9→9
3902 measured reflections	*l* = −18→18

### Refinement

**Table:**

Refinement on *F*^2^	Secondary atom site location: difference Fourier map
Least-squares matrix: full	Hydrogen site location: inferred from neighbouring sites
*R*[*F*^2^ > 2σ(*F*^2^)] = 0.036	H-atom parameters constrained
*wR*(*F*^2^) = 0.120	*w* = 1/[σ^2^(*F*_o_^2^) + (0.0795*P*)^2^ + 0.1078*P*] where *P* = (*F*_o_^2^ + 2*F*_c_^2^)/3
*S* = 1.05	(Δ/σ)_max_ < 0.001
2049 reflections	Δρ_max_ = 0.24 e Å^−^^3^
176 parameters	Δρ_min_ = −0.23 e Å^−^^3^
0 restraints	Extinction correction: *SHELXL97* (Sheldrick, 2008), Fc^*^=kFc[1+0.001xFc^2^λ^3^/sin(2θ)]^-1/4^
Primary atom site location: structure-invariant direct methods	Extinction coefficient: 0.036 (8)

### Special details

**Table:**

Geometry. All e.s.d.'s (except the e.s.d. in the dihedral angle between two l.s. planes) are estimated using the full covariance matrix. The cell e.s.d.'s are taken into account individually in the estimation of e.s.d.'s in distances, angles and torsion angles; correlations between e.s.d.'s in cell parameters are only used when they are defined by crystal symmetry. An approximate (isotropic) treatment of cell e.s.d.'s is used for estimating e.s.d.'s involving l.s. planes.
Refinement. Refinement of *F*^2^ against ALL reflections. The weighted *R*-factor *wR* and goodness of fit *S* are based on *F*^2^, conventional *R*-factors *R* are based on *F*, with *F* set to zero for negative *F*^2^. The threshold expression of *F*^2^ > σ(*F*^2^) is used only for calculating *R*-factors(gt) *etc*. and is not relevant to the choice of reflections for refinement. *R*-factors based on *F*^2^ are statistically about twice as large as those based on *F*, and *R*- factors based on ALL data will be even larger.

### Fractional atomic coordinates and isotropic or equivalent isotropic displacement parameters (Å^2^)

**Table:**

	*x*	*y*	*z*	*U*_iso_*/*U*_eq_	
C1	0.9386 (3)	0.64900 (17)	0.22991 (8)	0.0282 (3)	
C2	0.6877 (3)	0.75023 (17)	0.27294 (9)	0.0291 (3)	
H2	0.6254	0.8735	0.2630	0.035*	
C3	0.5297 (3)	0.66705 (18)	0.33088 (9)	0.0290 (3)	
C4	0.6221 (3)	0.48246 (18)	0.34697 (9)	0.0332 (3)	
H4	0.5166	0.4266	0.3858	0.040*	
C5	0.8736 (3)	0.38464 (17)	0.30388 (9)	0.0329 (3)	
H5	0.9361	0.2618	0.3152	0.040*	
C6	1.0383 (3)	0.46109 (17)	0.24430 (9)	0.0299 (3)	
C7	1.0249 (3)	0.90973 (18)	0.15901 (10)	0.0349 (3)	
H7A	1.0211	0.9696	0.2118	0.042*	
H7B	0.8424	0.9519	0.1412	0.042*	
C8	1.2360 (3)	0.94824 (18)	0.08886 (9)	0.0329 (3)	
C9	0.1228 (3)	0.70250 (18)	0.43152 (9)	0.0323 (3)	
H9A	0.2214	0.6572	0.4806	0.039*	
H9B	0.0869	0.6029	0.4068	0.039*	
C10	−0.1449 (3)	0.84177 (18)	0.46089 (9)	0.0321 (3)	
C11	1.3024 (3)	0.33765 (19)	0.20074 (10)	0.0363 (3)	
C12	1.4694 (4)	0.3989 (2)	0.12491 (12)	0.0524 (5)	
H12A	1.6153	0.2977	0.1007	0.079*	
H12B	1.5473	0.4845	0.1436	0.079*	
H12C	1.3527	0.4537	0.0818	0.079*	
O1	1.1504 (2)	1.10815 (14)	0.05426 (8)	0.0472 (3)	
H1	1.2707	1.1235	0.0164	0.071*	
O2	1.4614 (2)	0.84065 (14)	0.06836 (7)	0.0463 (3)	
O3	1.1036 (2)	0.72247 (13)	0.17277 (7)	0.0406 (3)	
O4	0.28549 (19)	0.77925 (13)	0.36867 (7)	0.0369 (3)	
O5	−0.2075 (2)	0.99593 (13)	0.43053 (7)	0.0421 (3)	
O6	−0.2994 (2)	0.78060 (14)	0.51955 (8)	0.0487 (3)	
H6	−0.4400	0.8616	0.5357	0.073*	
O7	1.3789 (3)	0.18327 (14)	0.22649 (9)	0.0579 (4)	

### Atomic displacement parameters (Å^2^)

**Table:**

	*U*^11^	*U*^22^	*U*^33^	*U*^12^	*U*^13^	*U*^23^
C1	0.0251 (6)	0.0269 (6)	0.0304 (6)	−0.0088 (5)	0.0037 (5)	0.0021 (5)
C2	0.0258 (7)	0.0232 (6)	0.0344 (7)	−0.0059 (5)	0.0025 (5)	0.0039 (5)
C3	0.0231 (6)	0.0288 (7)	0.0317 (7)	−0.0059 (5)	0.0019 (5)	0.0009 (5)
C4	0.0300 (7)	0.0300 (7)	0.0372 (7)	−0.0111 (5)	0.0041 (6)	0.0061 (5)
C5	0.0315 (7)	0.0233 (6)	0.0408 (8)	−0.0071 (5)	0.0005 (6)	0.0034 (5)
C6	0.0279 (7)	0.0247 (7)	0.0344 (7)	−0.0065 (6)	0.0003 (5)	−0.0003 (5)
C7	0.0299 (7)	0.0264 (7)	0.0420 (8)	−0.0064 (5)	0.0089 (6)	0.0039 (6)
C8	0.0306 (7)	0.0283 (7)	0.0375 (7)	−0.0101 (6)	0.0031 (6)	0.0028 (5)
C9	0.0266 (7)	0.0322 (7)	0.0342 (7)	−0.0087 (6)	0.0057 (5)	0.0036 (6)
C10	0.0292 (7)	0.0322 (7)	0.0328 (7)	−0.0105 (6)	0.0051 (5)	−0.0010 (5)
C11	0.0314 (7)	0.0283 (7)	0.0448 (8)	−0.0061 (6)	0.0033 (6)	−0.0045 (6)
C12	0.0432 (9)	0.0373 (8)	0.0602 (10)	−0.0011 (7)	0.0226 (8)	−0.0061 (7)
O1	0.0391 (6)	0.0365 (6)	0.0568 (7)	−0.0098 (5)	0.0118 (5)	0.0142 (5)
O2	0.0342 (6)	0.0394 (6)	0.0540 (7)	−0.0066 (5)	0.0142 (5)	0.0094 (5)
O3	0.0337 (6)	0.0259 (5)	0.0518 (6)	−0.0064 (4)	0.0185 (4)	0.0047 (4)
O4	0.0270 (5)	0.0298 (5)	0.0446 (6)	−0.0047 (4)	0.0126 (4)	0.0052 (4)
O5	0.0344 (6)	0.0333 (6)	0.0508 (6)	−0.0076 (4)	0.0102 (5)	0.0047 (4)
O6	0.0371 (6)	0.0384 (6)	0.0574 (7)	−0.0071 (5)	0.0228 (5)	0.0052 (5)
O7	0.0491 (7)	0.0285 (6)	0.0774 (9)	0.0037 (5)	0.0135 (6)	0.0050 (5)

### Geometric parameters (Å, º)

**Table:**

C1—O3	1.3596 (16)	C8—O2	1.2154 (18)
C1—C2	1.3862 (19)	C8—O1	1.3033 (17)
C1—C6	1.4170 (18)	C9—O4	1.4166 (15)
C2—C3	1.3884 (18)	C9—C10	1.4979 (19)
C2—H2	0.9300	C9—H9A	0.9700
C3—O4	1.3642 (17)	C9—H9B	0.9700
C3—C4	1.3949 (19)	C10—O5	1.2323 (17)
C4—C5	1.381 (2)	C10—O6	1.2863 (17)
C4—H4	0.9300	C11—O7	1.2126 (18)
C5—C6	1.3927 (19)	C11—C12	1.496 (2)
C5—H5	0.9300	C12—H12A	0.9600
C6—C11	1.4966 (19)	C12—H12B	0.9600
C7—O3	1.4079 (16)	C12—H12C	0.9600
C7—C8	1.5050 (18)	O1—H1	0.8200
C7—H7A	0.9700	O6—H6	0.8200
C7—H7B	0.9700		
			
O3—C1—C2	122.68 (11)	O2—C8—C7	122.72 (12)
O3—C1—C6	116.25 (11)	O1—C8—C7	112.58 (12)
C2—C1—C6	121.06 (12)	O4—C9—C10	109.47 (11)
C1—C2—C3	119.83 (11)	O4—C9—H9A	109.8
C1—C2—H2	120.1	C10—C9—H9A	109.8
C3—C2—H2	120.1	O4—C9—H9B	109.8
O4—C3—C2	114.79 (11)	C10—C9—H9B	109.8
O4—C3—C4	124.56 (12)	H9A—C9—H9B	108.2
C2—C3—C4	120.65 (12)	O5—C10—O6	124.81 (13)
C5—C4—C3	118.49 (12)	O5—C10—C9	122.91 (12)
C5—C4—H4	120.8	O6—C10—C9	112.28 (11)
C3—C4—H4	120.8	O7—C11—C12	119.44 (13)
C4—C5—C6	123.14 (12)	O7—C11—C6	118.94 (13)
C4—C5—H5	118.4	C12—C11—C6	121.60 (12)
C6—C5—H5	118.4	C11—C12—H12A	109.5
C5—C6—C1	116.82 (12)	C11—C12—H12B	109.5
C5—C6—C11	117.25 (11)	H12A—C12—H12B	109.5
C1—C6—C11	125.93 (12)	C11—C12—H12C	109.5
O3—C7—C8	106.80 (11)	H12A—C12—H12C	109.5
O3—C7—H7A	110.4	H12B—C12—H12C	109.5
C8—C7—H7A	110.4	C8—O1—H1	109.5
O3—C7—H7B	110.4	C1—O3—C7	119.30 (10)
C8—C7—H7B	110.4	C3—O4—C9	117.02 (10)
H7A—C7—H7B	108.6	C10—O6—H6	109.5
O2—C8—O1	124.70 (13)		
			
O3—C1—C2—C3	−179.66 (12)	O3—C7—C8—O1	163.06 (12)
C6—C1—C2—C3	−0.3 (2)	O4—C9—C10—O5	2.36 (19)
C1—C2—C3—O4	−179.26 (11)	O4—C9—C10—O6	−178.61 (11)
C1—C2—C3—C4	0.6 (2)	C5—C6—C11—O7	−9.3 (2)
O4—C3—C4—C5	179.78 (12)	C1—C6—C11—O7	171.32 (14)
C2—C3—C4—C5	−0.1 (2)	C5—C6—C11—C12	168.98 (14)
C3—C4—C5—C6	−0.8 (2)	C1—C6—C11—C12	−10.4 (2)
C4—C5—C6—C1	1.1 (2)	C2—C1—O3—C7	2.9 (2)
C4—C5—C6—C11	−178.34 (13)	C6—C1—O3—C7	−176.45 (12)
O3—C1—C6—C5	178.87 (12)	C8—C7—O3—C1	−176.25 (11)
C2—C1—C6—C5	−0.52 (19)	C2—C3—O4—C9	−176.96 (11)
O3—C1—C6—C11	−1.8 (2)	C4—C3—O4—C9	3.1 (2)
C2—C1—C6—C11	178.85 (12)	C10—C9—O4—C3	−175.88 (11)
O3—C7—C8—O2	−17.6 (2)		

### Hydrogen-bond geometry (Å, º)

**Table:**

*D*—H···*A*	*D*—H	H···*A*	*D*···*A*	*D*—H···*A*
O1—H1···O2^i^	0.82	1.83	2.6530 (14)	180
O6—H6···O5^ii^	0.82	1.83	2.6352 (15)	167
C2—H2···O7^iii^	0.93	2.44	3.3566 (17)	168
C4—H4···O6^iv^	0.93	2.52	3.4392 (19)	169

Symmetry codes: (i) −*x*+3, −*y*+2, −*z*; (ii) −*x*−1, −*y*+2, −*z*+1; (iii) *x*−1, *y*+1, *z*; (iv) −*x*, −*y*+1, −*z*+1.

## References

[bb1] Bruker (2004). *APEX2* and *SAINT* Bruker AXS Inc., Madison, Wisconsin, USA.

[bb2] Sheldrick, G. M. (2003). *SADABS* University of Göttingen, Germany.

[bb3] Sheldrick, G. M. (2008). *Acta Cryst.* A**64**, 112–122. 10.1107/S0108767307043930 18156677

[bb4] Spek, A. L. (2009). *Acta Cryst.* D**65**, 148–155. 10.1107/S090744490804362X PMC2631630 19171970

[bb5] Zhang, C., Li, H., Liu, D. & Liu, M. (2007). *Acta Cryst.* E**63**, o4210.

